# Water Assisted Growth of C_60_ Rods and Tubes by Liquid–Liquid Interfacial Precipitation Method

**DOI:** 10.3390/molecules17066840

**Published:** 2012-06-05

**Authors:** Hamid Reza Barzegar, Florian Nitze, Artur Malolepszy, Leszek Stobinski, Cheuk-Wai Tai, Thomas Wågberg

**Affiliations:** 1 Department of Physics, Umea University, Umea SE-901 87, Sweden; Email: hamid.barzegar@physics.umu.se (H.R.B.); florian.nitze@physics.umu.se (F.N.); 2 Faculty of Materials Science and Engineering, Warsaw University of Technology, Warsaw 02-507, Poland; Email: artur.mal7@wp.pl; 3 Institute of Physical Chemistry, Polish Academy of Sciences Kasprzaka 48/52, 01-224 Warsaw, Poland; Email: lstob50@hotmail.com; 4 Department of Materials and Environmental Chemistry and Berzelii Center EXSELENT on Porous Materials, Arrhenius Laboratory, Stockholm University, Stockholm S-106-91, Sweden; Email: cheuk-wai.tai@mmk.su.se

**Keywords:** fullerene, C_60_ rods, C_60_ tubes, LLIP method, water-ethanol mixture

## Abstract

C_60_ nanorods with hexagonal cross sections are grown using a static liquid–liquid interfacial precipitation method in a system of C_60_/*m*-dichlorobenzene solution and ethanol. Adding water to the ethanol phase leads instead to C_60_ tubes where both length and diameter of the C_60_ tubes can be controlled by the water content in the ethanol. Based on our observations we find that the diameter of the rods/tubes strongly depends on the nucleation step. We propose a liquid-liquid interface growth model of C_60_ rods and tubes based on the diffusion rate of the good C_60_ containing solvent into the poor solvent as well as on the size of the crystal seeds formed at the interface between the two solvents. The grown rods and tubes exhibit a hexagonal solvate crystal structure with *m*-dichlorobenzene solvent molecules incorporated into the crystal structure, independent of the water content. An annealing step at 200 °C at a pressure <1 kPa transforms the grown structures into a solvent-free face centered cubic structure. Both the hexagonal and the face centered cubic structures are very stable and neither morphology nor structure shows any signs of degradation after three months of storage.

## 1. Introduction

One dimensional fullerene structures (rods and tubes) in the nano- and sub-micrometer range are attractive materials due to their excellent properties suitable for applications in electronic devices, solar cells, optical switching devices, *etc*. [1,2,3,4,5,6,7,8,9,10]. The much higher abundance and better stability of C_60_ compared to other fullerenes [11] have attracted most interest into one-dimensional structures based on C_60_ molecules. However, although significant progress has been made during last years in obtaining better control over the properties of C_60_ one-dimensional structures, such as length, diameter and crystal structure [12,13,14], many challenges still exist both regarding the ability to fine tune these properties as well as understanding the growth mechanisms that allow tuning those properties.

There are several methods to produce one dimensional C_60_ structures including: slow evaporation [15,16], the use of templates [17], vapor-solid processes [18], fast solvent-evaporation techniques [19] and liquid-liquid interfacial precipitation (LLIP) [20,21,22,23]. Particular interest has been shown in the LLIP method because it involves several controllable parameters to affect the final product. In the LLIP method the C_60_ structures (rods and tubes) are formed at the interface between a good and a poor C_60_ solvent. The interface may either be influenced by hand shaking or ultra-sonication or left undisturbed during the synthesis process. The latter is referred to as static LLIP growth [24]. It has been reported that the morphology as well as the dimensions of the thus grown crystals can be affected by changing the involved solvents [14,19], changing the solvent ratio (ratio of the poor solvent to good solvent) [25], growth temperature [26] and by light illumination during the growth [27,28]. Recently Miyazawa and Hotta [24,25] have shown that the length of C_60_ nanowhiskers, synthesized by the hand shaking LLIP method, increased by adding water to isopropyl alcohol (IPA) in a system of IPA and C_60_-saturated toluene solution. However, in their experiments the water destabilizes the grown structures.Increasing the water content of IPA above a critical amount (2.3 mass%) only leads to granular C_60_ precipitates. Here we report on the influence of water on the growth of C_60_ structures synthesized by the static LLIP method, in a system of ethanol and C_60_*m*-dichlorobenzene (*m*-DCB). By increasing the water content of ethanol from 0 to 20 mass% we can tune the growth from C_60_ nanorods (100 nm in diameter and 1 mm in length) to C_60_ tubes (several micrometer in diameter and up to 1 cm in length). The structure and morphology of the grown tubes and rods are stable for more than three months under ambient conditions.

## 2. Results and Discussion

### 2.1. Morphology of the Grown C_60_ Structures

**Figure 1:**
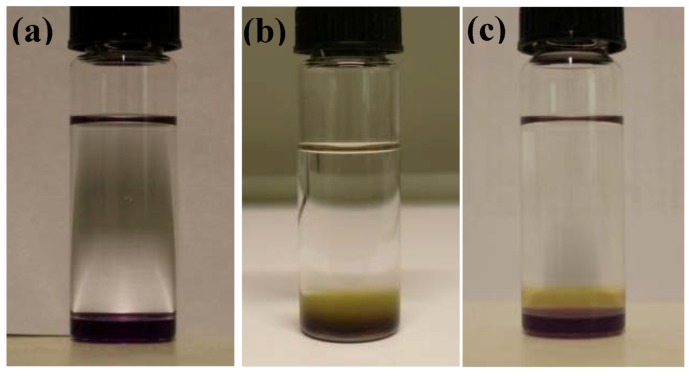
Photographs showing the interface by the static LLIP method for (**a**) C_60_ m-DCB solution and water-ethanol mixture; and (**b**) C_60_ m-DCB solution and pure ethanol after 3 h; (**c**) Sample shown in (**a**) but after ultra-sonication, three different phases are visible in the bottle.

**Figure 2:**
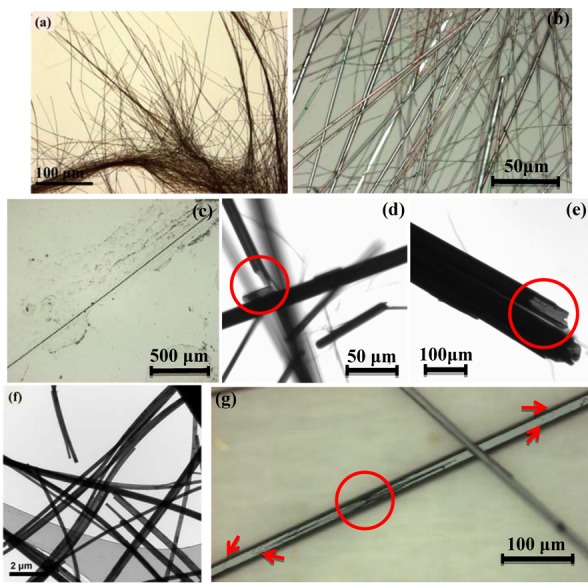
Optical micrograph of the grown C_60_ rods and tubes by using a water/ethanol ratio of (**a**) 0:100; (**b**) 2:100; (**c**) 5:100; (**d**) 10:100 and (**e**) 20:100. The visible tubular structures are marked by red circles. (**f**) TEM image of as-grown nanorods using a water/ethanol ratio of 0:100; (**g**) Optical micrograph of C_60_ tubes showing the void at the center of the tube corresponding to the nucleation site at the crystal seed. The growth fronts (marked with arrows) indicate the growth direction.

When growing one-dimensional C_60_ structures with the static LLIP method using an ethanol/*m*-DCB mixture as poor/good solvent and when adding water to the ethanol part of the mixture we observe growth times of 1 to 5 days. The growth process can be described as follows: after adding the water-containing ethanol to the C_60_/*m*-DCB solution an interface develops, as shown in Figure 1a. In the growth step, *m*-DCB diffuses into the ethanol-water mixture and the C_60_ rods/tubes start to grow at nucleation sites in the *m*-DCB/ethanol interface, as manifested by the formation of a growth region at the interface. The growth region is brownish and relatively wide in the case of pure ethanol (Figure 1b) but in the form of isolated black particles at the interface in the case of higher water content in the ethanol. The initial state is followed by a gradual disappearance of the lower pink phase. Simultaneously, brown or black C_60_ rods or tubes precipitates out of the solution and sink to the bottom of the beaker. By raising the water content of the ethanol we observe several distinct effects: (i) the growth time increases from 1 day for pure ethanol/*m*-DCB mixtures to 5 days for a water/ethanol ratio of 20:100; (ii) the *m*-DCB/ethanol-water interface becomes thinner, clearer, and more rigid at higher water content; (iii) at a water/ethanol ratio higher than 20:100 no growth occurs and the two phases of *m*-DCB and ethanol-water remain completely separated, even after a month; (iv) at higher water/ethanol ratios (but below 20:100) the rods grow with larger diameter and start to develop into tubes. This effect is clearly related to the water content and already at a water/ethanol ratio of 2:100 C_60_ tubes (with small diameter) starts to appear while not a single tube is found for pure ethanol. At higher water/ethanol ratios the C_60_ structures are predominantly tubes. Figure 2a–e show optical micrographs of C_60_ rods and tubes grown at different water/ethanol ratios.

From Figure 2a we can conclude that the nanorods synthesized using pure ethanol (without addition of water) are very flexible and tend to form bundles. Optical micrographs recorded with lower magnification revealed that the grown nanorods can be more than 1 mm in length.

**Figure 3:**
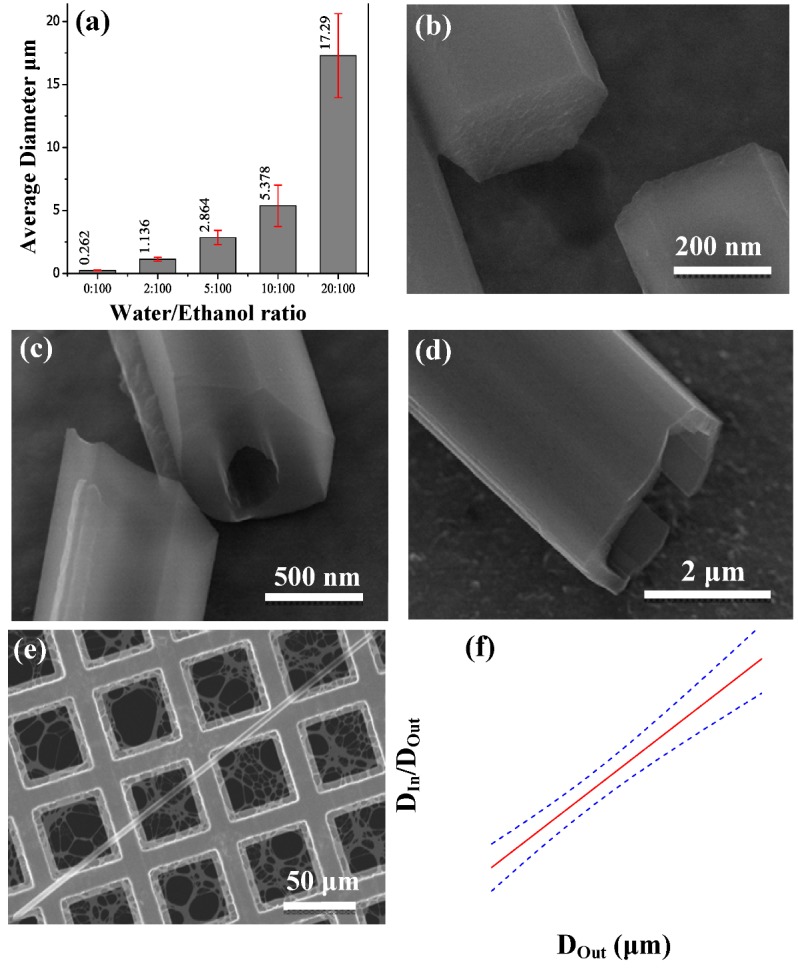
(**a**) Average diameter of the grown C_60_ structures at different water/ethanol ratios the error bars show standard error of mean; (**b**) SEM images indicating the hexagonal cross section of the C_60_ nanorods. SEM images of as-grown C_60_ tubes using a water/ethanol ratio of (**c**) 2:100; (**d**) 5:100; (**e**) 10:100; (**f**) A plot of the inner to outer diameter ratio as a function of the outer diameter, *i.e*., D_In_/D_Out_ *vs*. D_Out_.

The TEM and SEM micrographs, shown in Figure 2f and Figure 3a respectively, indicate that the nanorods have hexagonal cross sections and a diameter in the range of 100–400 nm, with an average diameter of 260 nm. Figure 2 shows that both diameter and length of the C_60_ structures increases as the water content in ethanol increases and that they reach a maximum diameter of 80 μm and about 1 cm in length for water/ethanol ratio of 20:100. A statistical analysis on the average diameter of the grown C_60_ structures at different water/ethanol ratio is shown in Figure 3a. The tubular C_60_ structures which develop when water is mixed into the ethanol are visible in optical micrographs 2d and 2e, marked by red circles, as well as in SEM images in Figure 3c–e. Figure 2g reveals that the tubes are very defective at the nucleation site (marked by red circle) before reaching a steady state growth. A strong indication for the direction of growth is a series of wave like patterns at the surface of the rods (indicated by arrows in Figure 2g) on both sides of the defective seed part.

The tubular morphology of the C_60_ structures grown by adding water to the ethanol is further supported by SEM images as shown in Figure 3c–e. An analysis of the wall thickness of the grown tubes, using SEM images, is presented in Figure 3f. The red line is a linear regression and the blue dashed lines show one sigma confidence bands. The plot reveals that the ratio of inner diameter to outer diameter (D_In_/D_Out_) increases linearly with increasing diameter of the C_60_ tubes.

Stability studies of our C_60_ rods and tubes, based on morphology and crystal structure measurements by TEM, SEM and XRD, show that even after three months there are no signs of degradation of the C_60_ structures. This is in contrast to earlier studies [24,25] where increasing the water content of isopropyl alcohol (IPA) above a critical value in a system of IPA and C_60_-saturated toluene solution destabilized the grown C_60_ nanowhiskers within less than a week. Our observations discussed above can be rationalized as follows: the solubility of C_60_ in ethanol is known to be very low (0.8 mg/L) [29]. In contrast, the high solubility of *m*-DCB in ethanol results in a fast diffusion of *m*-DCB molecules into the ethanol. Since the interface of *m*-DCB and ethanol is quickly over-saturated a large number of nano-sized C_60_ nucleation sites is provided (crystal seeds). Continued diffusion of *m*-DCB molecules into ethanol increases the concentration of C_60_ around the seeds which results in a growth of a large number of one dimensional C_60_ nanorods [13]. 

It has been suggested that water molecules can accumulate around alcohol chains and thereby affect the polarity of the water/ethanol cluster [30]. In addition, due to the low solubility of *m*-DCB in water [31] and the extreme hydrophobicity of C_60_, the addition of water to the ethanol efficiently slows down the diffusion of *m*-DCB molecules into the water/ethanol regions and thus decreases the rate at which the C_60_ molecules reach the oversaturation points. The combination of these two mechanisms leads to the formation of larger crystal seeds as well as more time for the C_60_ molecules to settle at the preferred circumferential edge sites which have been reported to have higher free energy compared to other sites [32,33]. Therefore, on the whole, increased water content leads to the growth of larger tubular C_60_ structures. This is in agreement with a previous report where the growth of C_60_ tubes is governed by the C_60_ concentration depletion around the crystal seed, resulting in growth only at the circumferential edges of the crystal seeds [13].

In order to study the influence of the nucleation step (crystal seed size) on the size of the grown C_60_ structures, the clear interface between the water/ethanol mixture and the C_60_ *m*-DCB solution was disturbed by ultra-sonication (as described in the Experimental section, see Figure 1c). The results show that, independent of the water content, the growth always leds to C_60_ nanorods similar to the ones obtained for the pure ethanol case as shown in Figure 2a. Due to ultra-sonication, the larger water/ethanol clusters are immediately scattered into a large number of small “micelles” where small crystal seeds are formed. Our results confirm that the diameter of the C_60_ rods is defined during the nucleation step and depends on the size of the crystal seeds. Although the diffusion rate of *m*-DCB into water/ethanol mixtures is slow and the growth time was long, we did not observe any tubular structures when the interface was ultra-sonicated. This indicates that the growth of C_60_ tubes is not solely depending on the diffusion rate of C_60_ molecules at the interface, but also depends on the diffusion length of C_60_ at the surface of crystal seeds, as suggested by Mayer and Xia [32]. This means that formation of tubular structures can only occur when the size of the crystal seed is larger than the diffusion length of C_60_ molecules. This explains both our observation that no tubular structures are observed for ultra-sonication, small seeds respectively, as well as the increase of D_In_/D_Out_ for tubes with larger diameters. 

### 2.2. Composition and Crystal Structure of C_60_ Rods and Tubes

The chemical composition and structure of the C_60_ tubes and rods were characterized by a number of different techniques. We observe that the differences in morphology for different growth parameters are not expressed as a difference in chemical composition or structure. All results below are equally valid for all rod and tube types.

#### 2.2.1. Thermogravimetric Analysis

**Figure 4:**
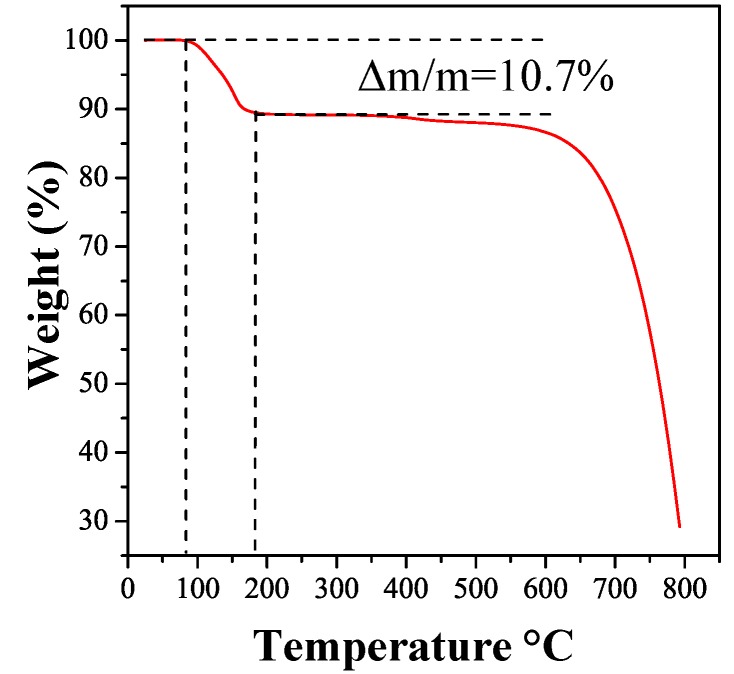
TGA result recorded on as-grown rods at a heating rate of 5 °C/min in Ar flow.

Figure 4 shows a TGA measurement performed on the as-grown C_60_ structure. The result shows a clear weight loss of 10.7% in a temperature range between 85 °C to 171 °C (close to the evaporation temperature of *m*-DCB) which can be assigned to the evaporation of the *m*-DCB molecules from the tubes structure. Based on the TGA result a 24 h heat treatment at 200 °C in vacuum (*p* < 1 kPa) could be considered to result in a complete removal of solvent molecules from C_60_ structures.

#### 2.2.2. FT-IR Spectroscopy

**Figure 5:**
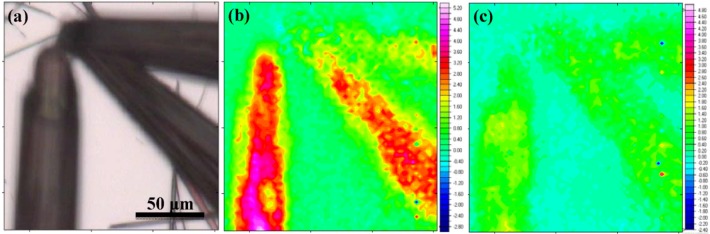
FT-IR spectroscopy results, (**a**) optical image of the sample; (**b**) A C_60_ trace map based on the intensity of the C_60_ characteristic peak at 1182 cm^−1^; (**c**) A m-DCB trace map based on the intensity of the m-DCB characteristic peak at 1462 cm^−1^. The units on the y-scale in (**b**) and (**c**) arearbitrary.

Figure 5 presents the results of FT-IR spectroscopy performed on the sample synthesized with a water/ethanol ratio of 10:100. Figure 5b,c displays selective FT-IR mapping with respect to the intensities of the characteristic C_60_ vibration (at 1182 cm^−1^) and *m*-DCB vibration (at 1462 cm^−1^) respectively. A comparison of Figure 5b,c with the optical micrograph of the sample in Figure 5a reveals that the as-grown rods are composed of C_60_ and *m*-DCB molecules indicating that solvent molecules are incorporated in the C_60_ rod structures in agreement with the TGA data and with earlier reports [19]. The weak signal from the upper tube in Figure 5b,c is due to the fact that it is slightly out of focus, as can be seen in Figure 5a. The trace of *m*-DCB was still observable in the sample that had been annealed at 150 °C, but disappears completely after the annealing step at 200 °C (data not shown).

#### 2.2.3. Raman Spectroscopy

**Figure 6:**
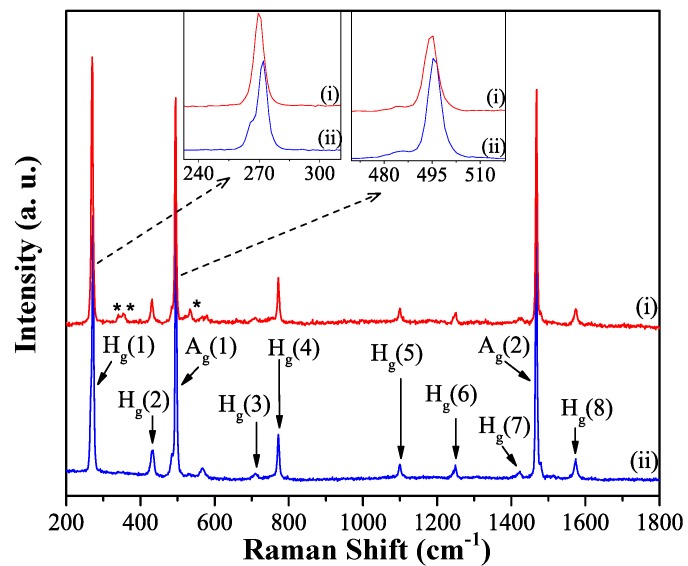
Raman spectra recorded on as-grown rods (upper red trace i) and pristine C_60_ (lower blue trace ii). Asterisks indicate the peaks which are assigned to the incorporation of m-DCB in the C_60_ rod/tube structure. Insets show enlarged regions around the down-shifted A_g_ (1) and H_g_ (1) modes due to the presence of m-DCB in the rod/tube.

The incorporation of *m*-DCB molecules in the structure of as-grown C_60_ rods was further confirmed by Raman spectroscopy. Figure 6a displays typical Raman spectra of the as-grown rods (upper red spectrum i) together with the pristine C_60_ powder (lower blue spectrum ii). Ten characteristic Raman vibrational modes of C_60_ (8 H_g_ and 2 A_g_) are observed in both spectra. Besides the characteristic C_60_ peaks, three additional peaks, positioned at 341, 353 and 535 cm^−1^ (marked with asterisks) are present in the spectrum of as-grown rods. These peaks disappear after the annealing step at 200 °C and are therefore assigned to the presence of m-DCB molecules in the rods. The more thorough comparison of two spectra in Figure 6 also indicates a downshift of the A_g_ (1) and H_g_ (1) modes in the spectrum of as-grown C_60_ rods, from 272 and 496 cm^−1^ to 269 and 494 cm^−1^ respectively, (see insets in Figure 6). This down shiftis not observed in the spectrum of the annealed rods which is identical to that of pristine C_60_ (data not shown). Earlier Raman studies on crystalline C_60_-solvate structures show a similar down shift and were attributed to interaction between C_60_ and solvent molecules [15,19,34].

The Raman peak at 1468 cm^−1^ corresponding to the A_g_ (2) pentagonal pinch mode of C_60_ is known to be strongly sensitive to charge transfer and intermolecular bonding [35,36,37,38,39]. Our analysis reveals that the A_g_ (2) mode of the as-grown rod (Figure 6) is similar to the one in pristine C_60_ both regarding shape and position. This suggests that the C_60_ molecules in rod/tube structures are in the monomeric state and there is no C_60_ polymerization in the as-grown rods. 

#### 2.2.4. X-ray Diffraction Pattern

**Figure 7:**
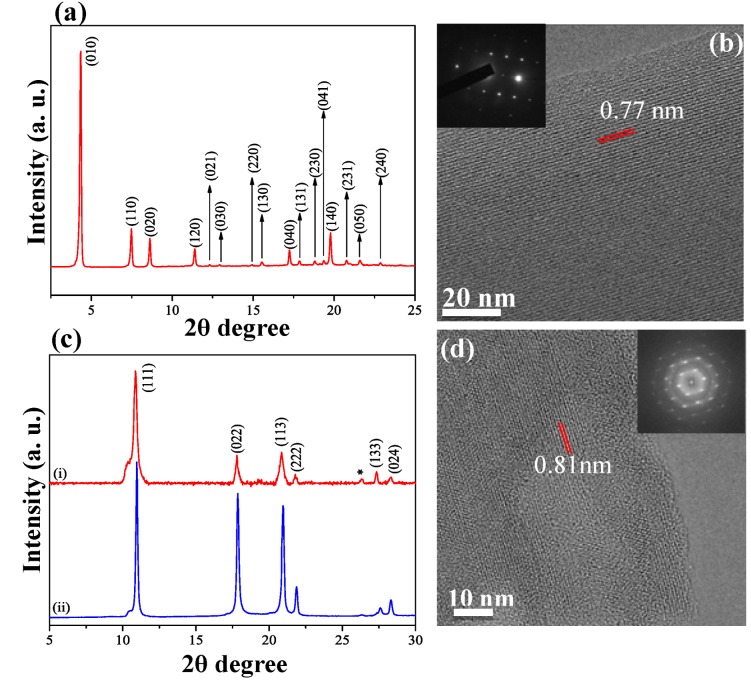
(**a**) XRD pattern of as-grown rods indicating ahcp structure; (**b**) Typical HRTEM image of as-grown rods. The inset shows the SAED pattern of the rod; (**c**) XRD pattern of the annealed rods (upper red trace i) and pristine C_60_ (lower blue trace ii); (**d**) HRTEM image of annealed rods, grain boundaries are visible in the image. The inset shows the corresponding FFT pattern, the streaks reveal the presence of grain boundaries and their direction are well known to run perpendicular to the grain boundaries. The asterisks in the 7(**c**) shows the peaks from the used substrate (silicon wafer).

Figure 7a presents a typical XRD pattern of the as-grown rods. The pattern can be well assigned to a hcp crystal structure with a unit cell size of *a = b =* 23.71 Å and *c =* 10.18 Å. Figure 7b shows a typical high-resolution TEM (HRTEM) image of an as-grown rod, indicating the (020) lattice planes with a lattice plane spacing of 0.77 nm. The inset in Figure 7b shows the corresponding selected area electron diffraction (SAED) pattern. The combination of clear lattice fringes and sharp and distinct diffraction spots reveal that the as-grown rods are perfectly crystalline. By examining the entire rod length with HRTEM and SAED, we find that the full length of the rods is indeed a single crystal. Although mentioned above that all results are equally valid for both tubes and rods, we would like to point out that all rods and tubes are highly crystalline independently of their diameter. This is partly contradicting earlier reports which stated that only rods can grow as single crystals [18]. Figure 7c shows the XRD patterns of annealed rods (upper red pattern i) together withpristine C_60_ (lower blue pattern ii). By removing the solvent molecules from the crystal the structure transforms to a fcc structure known to be characteristic for pristine C_60_ [20,40]. The diffraction peaks in Figure 7c are all assigned to the fcc crystal structure and corresponds to a unit cell size of *a = b = c =* 14.13 Å. Figure 7d shows a typical HRTEM image of the annealed rods with a lattice plane spacing of 0.81 nm corresponding to the lattice planes (111). Distinct grain boundaries are visible in the image which indicates that the annealing step, which evaporates the solvent molecules, transforms the structure from single crystalline to crystalline regions where the lattice planes are slightly disoriented relative to each other. This is even clearer by the streaks in the Fast Fourier Transform (FFT) diffractogram pattern of the HRTEM images (inset in Figure 7d), which are well known to run perpendicular to the grain boundaries, and also by the broadening of the peaks in the XRD pattern of the annealed rods compare to the pure C_60_.

**Figure 8:**
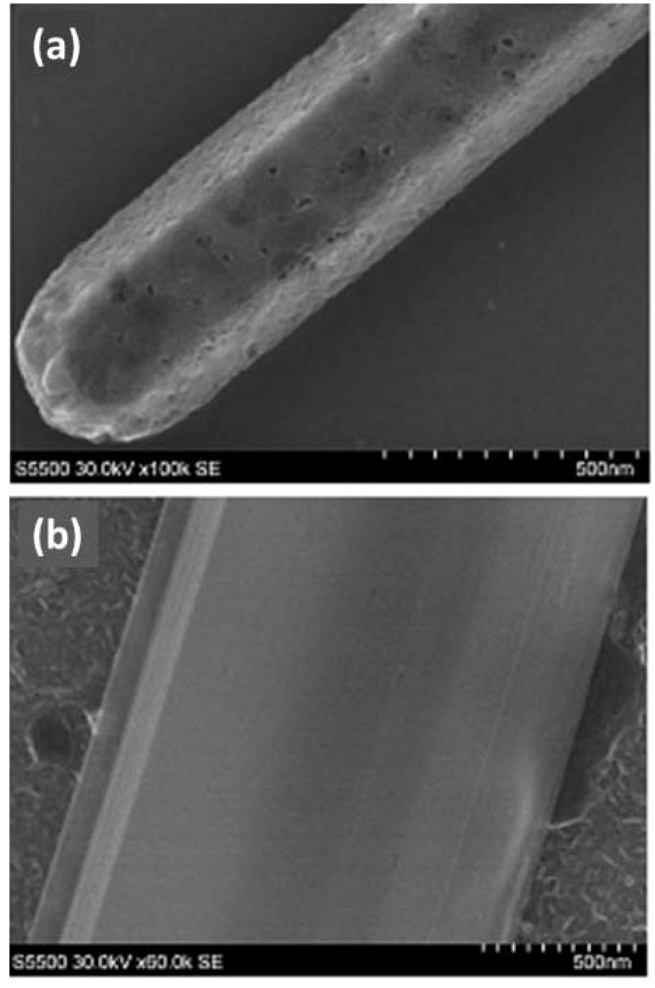
SEM images of annealed rods and tubes. (**a**) Typical SEM image of annealed nanorods where the pores, resulting from the annealing, are very pronounced; (**b**) Typical SEM image of annealed C_60_ tubes which clearly contain less number of pores and the few which exist are also smaller.

SEM images of the annealed structures (Figure 8) indicate that the annealing step, resulting in removal of solvent molecules, also introduces an element of porosity into the structure. By comparing the SEM and HRTEM images of different samples we found that the “porosity” is less pronounced in C_60_ tubes than in C_60_ rods. This could be explained by the fact that C_60_ tubes have higher exposed surface area compare to C_60_ rods and thus incorporated solvent molecules can easier escape from such structures.

## 3. Experimental

### 3.1. C_60_ Rod and Tube Synthesis

The C_60_ powder (>99.9%, MER Corporation) was first degassed at 150 °C for 12 h in vacuum (10^-2^ mbar) to remove any possible oxygen in the structure, and then dissolved in *m*-dichlorobenzene (m-DCB >99.0%, Sigma-Aldrich) by ultra-sonication for 45 minutes. A stock C_60_/m-DCB solution was prepared with a concentration of 1 mg/mL. Ethanol (99.5%, Kemetyl) with a maximum water content of 0.5% was used as the poor solvent of C_60_.

In this work we use the term C_60_ nano-rod/tube when the diameter of the grown rod/tubes is smaller than 500 nm and structures with diameter larger than 500 nm are referred to C_60_ rod/tubes. The C_60_ rods and C_60_ tubes were synthesized by the LLIP method as follows; 10 mL of ethanol was first mixed with distilled water (with the desired ratio). After hand shaking, the mixture was gently added to 1 mL of C_60_/m-DCB solution (in a 15 mL transparent glass bottle), so that a clearly defined interface between the two solutions could be observed. Two different procedures were tested and evaluated. In process (i) the bottle was tightly closed and stored at room temperature for 1 week without any disturbance (see Figure 1a). In process (ii) the blend solution was ultra-sonicated for 10 s in an ultrasonic bath, (USC300D from VWR) in such way that three distinguishable phases were formed in the bottle as shown in Figure 1c and then the bottle was stored at room temperature with a tightly closed cap for 1 week. For both processes C_60_ rods and tubes were prepared using five different volume ratios of water to ethanol: 0:100, 5:100, 10:100 and 20:100 (the original water content, 0.5%, of the used ethanol was not considered in the calculation of the water ethanol ratios).

Due to the diffusion of the solvent the interface disappears and C_60_ structures start to grow. The growth continues until the lower phase in the bottle completely disappears. In this work the time between creating the interface and disappearing of the lower phase is considered as a ‘growth time’.

### 3.2. Sample Characterization

In order to check the stability of the crystal structure and morphology of the grown C_60_ structure, the characterization of the samples was done within an interval of three months after the growth, where the samples were kept in their original bottles.

The C_60_ rods/tubes were characterized by X-ray diffraction (XRD, Siemens D5000 diffractometer, wavelength (Cu K_α_) = 1.5418 Å, accelerating voltage = 40 kV), Fourier-transform infrared spectroscopy (FT-IR, Tensor 27 FT-IR microscope), Raman spectroscopy (in Via Raman Microscope Renishaw, excitation wavelength = 785 nm), transmission electron microscopy (TEM, JEOL 1230 and JEOL 2100F, accelerating voltage = 80 kV and 200 kV, respectively), field-emission scanning electron microscopy (SEM, Hitachi S-5500 In-lens high resolution FE-SEM), thermal gravimetric analysis (TGA, Mettler Toledo TGA/DSC 1 LF/948, heating rate = 5 °C/min, measurement under Ar flow), and optical microscopy (Olympus BX51 equipped with an Infinity 2-1C CCD camera). The samples were deposited on the substrate of choice (silicon wafer for XRD, FT-IR, Raman spectroscopy and glass substrate for optical microscopy), followed by drying at room temperature. For the TEM and SEM measurements, the samples were loaded on a TEM supporting grid with holey carbon film by dipping the grid into the sample dispersion. The annealing of the samples took place on the substrate of choice by using a vacuum oven (Jeio Tech, model OV-11) for 24 h at *T* = 200 °C and *p* < 1 kPa.

## 4. Conclusions

C_60_ nanorods were grown using the LLIP method in a combination of C_60_ m-DCB solution and ethanol. It is suggested that the addition of water to ethanol increases the size of the crystal seeds and decreases the diffusion rate of m-DCB (and thereby the diffusion rate of C_60_) into the water/ethanol cluster. We can explain the growth of C_60_ tubes based on these two properties in combination with the diffusion length of C_60_ molecules on the crystal seed. By increasing the water content of the ethanol, C_60_ tubes with larger width, up to 80 μm, and longer length, up to 1 cm were grown. The addition of water only affects the morphology of the grown rods and tubes but they have similar chemical composition and crystal structure independent of the water content. Our method suggests that controlling the nucleation step (size of the crystal seed) in combination with the diffusion rate of the good solvent into the poor solvent are key points in controlling the size and morphology of the C_60_ structures. By this knowledge our study improves the possibility to investigate size dependent properties of one dimensional C_60_ structures.

## Supplementary Materials

Supplementary File 1
