# Supplementary material for: Water Assisted Growth of C60 Rods and Tubes by Liquid–Liquid Interfacial Precipitation Method
Source: Molecules. 2012 Jun 5;17(6):6840–53. doi: 10.3390/molecules17066840 (PMC6268927; doi:10.3390/molecules17066840)

Note:

Figure 3f is not displayed correctly in the PDF file, which is missing its axis. A correct figure is listed below, and for proper appearance of the figures we recommend to download the WORD file of this paper:

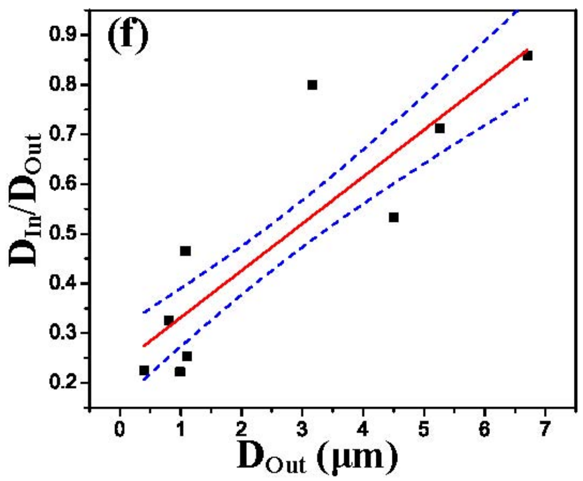

Supplement: Supplementary File 1 [file molecules-17-06840-s001.pdf]
